# Therapeutic Potential of Bone Marrow- and Ovarian/Endometrium-Derived Mesenchymal Stem Cells in Regulating Ovarian Function in a Streptozotocin-Induced Diabetes Mellitus Rat Model

**DOI:** 10.1007/s43032-026-02063-1

**Published:** 2026-03-05

**Authors:** Eda Tunç, Naz Dizeci, Ferda Alpaslan Pınarlı, Özlem Yıldırım

**Affiliations:** 1 Faculty of Science, Department of Biology, Molecular Biology, Ankara University, Tandogan, Ankara, 06100 Turkey; 2 Faculty of Medicine, Department of Medical Biology, Ankara Medipol University, Anafartalar, Ankara, Altındag 06050 Turkey; 3 Viagen Laboratory and Health Services, Çankaya, Ankara, Turkey

**Keywords:** Streptozotocin-induced diabetes mellitus, Ovarian, Bone marrow, Mesenchymal stem cell, Immunohistochemistry, Follicle maturation, Infertility

## Abstract

Streptozotocin (STZ)-induced pancreatic damage leads to β-cell injury and hyperglycemia, providing a controlled model to study ovarian dysfunction. This study aimed to investigate the potential of mesenchymal stem cells (MSCs) derived from ovary, endometrium, and bone marrow to restore ovarian function in rats with STZ-induced diabetes mellitus, due to their ease of isolation and capacity for differentiation. Fifty female Sprague-Dawley rats were divided into five groups: control, STZ-induced pancreatic damage, and STZ-induced rats treated with ovarian, endometrium, or bone marrow-derived MSCs. Pancreatic damage was induced via intraperitoneal injection of 45 mg/kg STZ in 32 rats. Rats with blood glucose ≥ 200 mg/dL were considered hyperglycemic and monitored for six weeks. MSCs were isolated from healthy donors, cultured, characterized by flow cytometry, and labeled with BrdU. At the third week, 2 × 10⁶ cells in PBS were injected into the ovaries via the endometrial canal. At the sixth week, rats were sacrificed, and ovarian tissues were analyzed histopathologically and immunohistochemically. The results showed that streptozotocin-induced diabetes mellitus led to structural alterations in ovarian follicles. Notably, bone marrow- and ovary-derived MSCs were effective in ameliorating these changes, while endometrium-derived MSCs showed moderate effects. These findings suggest that MSCs have therapeutic potential to mitigate ovarian dysfunction caused by pancreatic β-cell injury and hyperglycemia, highlighting their possible role as a cellular therapy in models of STZ-induced ovarian impairment.

## Introduction

Diabetes is a chronic metabolic disorder characterized by hyperglycemia resulting from defects in insulin secretion or insulin action [1]. As a progressive disease, it can lead to acute and chronic complications if not properly controlled. The persistent elevation of glucose levels in the blood has toxic effects and can cause varying degrees of damage to all cells in the body. Although this damage progresses slowly, it is highly dangerous [2]. The tissue and organ damage caused by hyperglycemia over time can far exceed the destructive effects of many diseases that act over a shorter period. It is a disease that advances with defects in carbohydrate, protein, and fat metabolism, accelerated arteriosclerosis, and changes in capillary membranes [3]. The most common chronic complications of diabetes mellitus include retinopathy, nephropathy, foot ulcers, neuropathy, gastrointestinal and sexual dysfunction, and cardiovascular syndromes [3].

The effects of insulin on carbohydrate metabolism are well known, but this hormone also plays a significant role in regulating ovarian function. A wide range of reproductive abnormalities may occur in individuals with type 1 diabetes due to insulin deficiency or excess. Insulin enhances the growth of pre-ovulatory follicles through its gonadotropic effect [4], reduces apoptosis and atresia of ovarian follicles [5], and stimulates the transition from primordial to primary follicles [6]. Insulin deficiency may lead to lower gonadotropin levels due to decreased GnRH secretion. Hyperglycemia can affect the ovary both directly and by inducing insulin resistance caused by glucose toxicity. Elevated serum insulin levels may result in the over stimulation of insulin and IGF-1 receptors in the ovary, increase steroid secretion, and promote the development of PCOS [7]. Since diabetes affects vascular and nerve function, it can induce structural and functional changes in the female genital organs, thereby impacting sexual response. Various animal studies have shown that diabetes reduces sexual desire and orgasm sensation [8], decreases the muscle layer and epithelial thickness of the vagina, and leads to vaginal fibrosis by reducing blood flow [9]. In diabetic women, ovarian functions are impaired, leading to reduced ovulation and fertility rates. Consequently, decreased ovarian reserves have been reported [10]. Studies have also revealed increased follicular and stromal degeneration, as well as stromal fibrosis, in the ovarian tissues of diabetic rats compared to normal rats. Furthermore, the normal structure of the ovary was disrupted, and stromal inflammation was observed in diabetic rats [11].

There are various treatment methods for diabetes, and in recent years, cell therapies have brought new hope. Stem cells are a significant resource for the treatment of diabetes because they can differentiate into insulin-producing cells [12–14]. Adult stem cells tend to differentiate primarily into the cell types of the tissue from which they originate, unlike embryonic stem cells that have broader pluripotency (e.g., mesodermal, endodermal, and ectodermal lineages [15, 16]. Furthermore, mesenchymal stem cells (MSCs) contribute to tissue protection and regeneration largely through secretion of paracrine factors—such as cytokines, growth factors, chemokines, and extracellular vesicles—which modulate immune responses, prevent apoptosis, and enhance survival and proliferation of neighboring cells, including pancreatic β-cells [17, 18]. When the selected cytokines and growth factors known for their protective effects, such as TGFβ and IL-6, were labeled with antibodies, they were found to be more intensely positive in the cells of the graft area [19]. The use of mesenchymal stem cells in regenerative and reparative therapies is currently widely studied in research [20–21].

This study aimed to investigate the therapeutic potential of mesenchymal stem cells (MSCs) in restoring ovarian function in rats with streptozotocin (STZ)-induced diabetes mellitus. Considering the ease of isolation and the capacity of MSCs to differentiate into epithelial cells, the research focused on the effects of ovary-derived, endometrium-derived, and bone marrow-derived MSCs on ovarian structure and function in this model. By administering MSCs to different treatment groups, the study assessed whether MSC therapy could ameliorate ovarian alterations caused by STZ-induced β-cell injury and hyperglycemia, providing insights into potential strategies for mitigating ovarian dysfunction in the context of pancreatic damage.

## Materials and Methods

### Experimental Animal Study

All experimental procedures were conducted at the Stem Cell Research Center affiliated with Yıldırım Beyazıt Training and Research Hospital (Ankara, Türkiye). The study protocol was approved by the Ethics Committee of Yıldırım Beyazıt Training and Research Hospital and was carried out in accordance with the Guide for the Care and Use of Laboratory Animals and the European Parliament Directive 2010/63/EU.

Fifty female Sprague-Dawley rats, eight weeks old and weighing between 150 and 180 g, were used in the experiment. The animals were randomly assigned to five experimental groups (*n* = 8 per group). In addition, 10 female rats of the same age and weight range were allocated specifically for the isolation of mesenchymal stem cells from fetal tissue (gestational day 16). The experimental groups were as follows:

1. Control Group (*n* = 8): Healthy rats without any intervention.
2. Diabetes Group (*n* = 8): Rats induced with diabetes via a single intraperitoneal injection of streptozotocin (STZ, 45 mg/kg).
3. OMSC Treatment Group (*n* = 8): Diabetic rats treated with ovary-derived mesenchymal stem cells.
4. EMSC Treatment Group (*n* = 8): Diabetic rats treated with endometrium-derived mesenchymal stem cells.
5. BMMSC Treatment Group (*n* = 8): Diabetic rats treated with bone marrow-derived mesenchymal stem cells.

The rats were housed under controlled environmental conditions at a temperature of 20–24 °C, with 40–60% relative humidity, and maintained on a 12-hour light (06:00–18:00) and 12-hour dark cycle. All animals were fed a standard rat chow diet and had ad libitum access to water. They were kept in group-housed cages with clean bedding material, and every effort was made to minimize discomfort and pain throughout the study.

### Induction of Experimental Diabetes Model

To induce diabetes, 32 female Sprague-Dawley rats were administered STZ (Sigma-Aldrich) at a dose of 45 mg/kg, dissolved in 0.1 mmoL sodium-citrate buffer (pH 4.5), via intraperitoneal injection. To confirm the development of diabetes, blood glucose levels were measured from tail vein blood samples 72 h after injection, and rats with blood glucose levels of 200–250 mg/dL or higher were considered diabetic. The diabetic rats were monitored for 6 weeks, with blood glucose levels and body weights measured and recorded at the 72nd hour, 3rd week, and 6th week, both in the morning and evening [22].

### Mesenchymal Stem Cell Isolation

Following deep ketamine/xylazine anesthesia, the abdominal area of the Sprague-Dawley rats (gestational day 16) was cleaned with Betadine^®^ (10% povidone-iodine solution; Mundipharma), an antiseptic commonly used for skin disinfection prior to surgical procedures, and neonatal ovarian and endometrial tissues were collected. Immediately after collection, the tissues were placed into a transport medium and transferred without delay to the laboratory. Upon arrival, the tissues were placed in a glass Petri dish and cut into small pieces using a scalpel. They were then digested into a single-cell suspension using 1 mg/mL collagenase type I (Sigma, Missouri, United States) and transferred to the bottom of a T25 flask. After incubating for 15–30 min, the tissue digests were sequentially filtered twice through 100 μm cell strainers (Corning, New York, United States) and then through a 40 μm cell strainer (Corning), effectively removing undigested tissue and epithelial cells at each step. Finally, mesenchymal stem cell culture medium was gently added. The culture medium was changed every three days, and passaging was performed based on cell growth and development [23–24].

Under deep ketamine/xylazine anesthesia, the 16-day-old fetal Sprague-Dawley rats designated for mesenchymal stem cell isolation were euthanized. The abdominal region was disinfected with Betadine^®^ (10% povidone-iodine solution), and neonatal bone marrow material was collected from the femurs and tibias into a transport medium immediately after extraction. Both ends of each bone were fractured under sterile conditions, and the marrow cavity was flushed using 5 mL syringes filled with mesenchymal stem cell culture medium. The bone marrow suspension was collected into sterile Falcon tubes. Ficoll was added to new Falcon tubes, and the collected bone marrow was slowly layered onto the Ficoll solution. The samples were centrifuged at 1200 rpm for 5 min. The resulting buffy coat was collected into new tubes, and the volume was adjusted to 10 mL with mesenchymal stem cell culture medium. The samples were centrifuged twice at 1200 rpm for 5 min each time. The supernatant was discarded, and fresh mesenchymal stem cell culture medium was added to the cell pellet. The cells were seeded into T25 flasks. The culture medium was changed every three days, and passages were performed based on cell growth and confluence [24–25].

The stem cells isolated during the experimental process were cultured in Dulbecco’s Modified Eagle’s Medium (DMEM) containing 10% (v/v) heat-inactivated fetal bovine serum (FBS), 20 mM L-glutamine, and 1% penicillin-streptomycin.

### Characterization of Mesenchymal Stem Cells Using Flow Cytometry

In this study, the flow cytometry method was utilized to determine the differentiation potential of primary cells into multipotent cell types such as osteoblasts, chondrocytes, and adipocytes. For this purpose, cells were suspended after the second passage to a concentration of 1 × 10⁶ cells in 100 µL aliquots, and 10 µL of surface markers (CD45, CD11b/c, CD44, CD90; all markers from BD-USA) were added and vortexed. The samples were incubated in the dark at 4 °C for 15 min. Following incubation, 2 mL of washing solution was added, and the samples were centrifuged at 1200 rpm for 10 min, after which the supernatant was discarded. To the cell pellet, an additional 1 mL of washing solution was added, and the samples were characterized using a flow cytometry device (FACS ARIA III, Becton, Dickinson and Company, Vancouver, Canada) [24, 26].

### Characterization of Mesenchymal Stem Cells by Tri-lineage Differentiation Assay

To confirm differentiation into adipocyte cells, the cells were cultured for three weeks using Adipocyte Differentiation Basal Medium and supplements (Gibco, USA), and lipid droplets were visualized in red using the Oil Red staining method (Diagnostic BioSystem, USA). To determine differentiation into chondrocyte cells, Chondrocyte Differentiation Basal Medium was utilized, and the cells were cultured for three weeks, with the presence of hyaluronic acid detected by staining with Alcian Blue (Diagnostic BioSystem, USA), resulting in a blue coloration. The differentiation of osteocyte cells was assessed by culturing them with Osteocyte Differentiation Basal Medium, followed by the Von Kossa staining method (Diagnostic BioSystem, USA), which stained calcium deposits black [27–29].

### Application of Stem Cells in Diabetic Rats

To track the localization of stem cells administered to diabetic rats at the end of the experiment and to evaluate their contribution to healing, mesenchymal stem cells were stained with BrdU (Bromodeoxyuridine). For the staining procedure, a 10 µM BrdU solution was prepared at a concentration of 1 mL. The cells were incubated for 2 h (37 °C, 5% CO₂) by adding 10 µL of the BrdU solution to each 1 mL containing 2 × 10⁶ cells. The identified mesenchymal stem cells were labeled with BrdU and injected into diabetic rats induced with STZ at the end of the third week via the endometrial canal to the ovary, at a concentration of 2 × 10⁶ cells/mL PBS in a total volume of 1 mL. This procedure was repeated in the fourth week. At the end of the sixth week, the abdominal cavities of the rats were surgically opened, the ovarian tissues were excised, and fixation was performed using 4% formaldehyde. For histological and immunohistochemical analyses, all rat groups were anesthetized via intraperitoneal injection of ketamine and xylazine. The abdominal cavities of the rats were opened, and the ovarian tissues were excised and fixed in 4% formaldehyde. The fixed tissues were dehydrated using increasing concentrations of ethanol. Subsequently, the tissue was embedded in melted paraffin at 60 °C, and blocks were prepared. The prepared blocks were sliced into 4 μm thick sections using a microtome [24, 30].

### Histochemical Analyses Using Hematoxylin & Eosin (H&E) Staining

Sections with a thickness of 4 μm obtained from paraffin blocks were subjected to routine histological procedures. For the deparaffinization stage, the sections were incubated overnight at 37 °C, followed by an additional hour at 57 °C. After this process, the sections were placed in xylene three times for 20 min each and then rehydrated in a series of decreasing ethanol concentrations (100%, 90%, 80%, 70%, and 50%), each for 10 min. Following air drying, the sections were washed with tap water for 10 min to remove any residual ethanol. Subsequently, the sections were incubated in Harris hematoxylin staining solution for 10 min, rinsed again under tap water for 10 min, and immersed 2–3 times in a mixture of glacial acetic acid and ethanol before being washed under tap water for another 10 min. Following this, the sections were treated in Eosin staining solution for 10 min and then washed again with tap water for 10 min. The dehydration process was completed by quickly passing the sections through increasing concentrations of alcohol (50%, 70%, 80%, 90%, and 100%). Finally, the sections were treated with xylene for 15 min and mounted with Entellan. The images obtained from the prepared sections were captured using a Leica DM 4000B (Germany) computer-assisted light microscope and evaluated using the Leica LAS V4.9 software [31].

### Immunohistochemical Analyses

The immunohistochemical method was performed using the Ultra Vision Detection System Large Volume Anti-Polyvalent, HRP (RTU) and the Ultra Vision Detection System DAB Substrate kits. For immunohistochemical staining, 4 μm thick sections were taken from paraffin blocks and placed on polylysine-coated slides. After the deparaffinization stage, the sections were processed through a series of decreasing alcohol concentrations of 100%, 96%, 90%, 80%, and 70%. The dehydrated tissues were rinsed twice for 5 min with distilled water to remove the ethanol, followed by antigen retrieval using citrate buffer (pH 6.0). To remove the citrate buffer, the tissues were washed twice again with distilled water for 5 min each. The sections were then placed in an immunohistochemistry chamber surrounded by a PAP pen to maintain a moist environment. The tissues were washed three times for 3 min each with phosphate-buffered saline (PBS, pH 7.4), and then treated with 3% hydrogen peroxide for 15 min. After this treatment, the slides were washed with PBS and incubated with UltraV block (TA-125-UB, Lab Vision, Fremont, CA) for 5 min to prevent nonspecific binding. Without washing, the primary antibody application was performed, and the sections were incubated overnight with VEGF primary antibody (bs-1665R, Bioss, USA) at an appropriate dilution (1:200). Additionally, for stem cell labeling, the sections were incubated for 60 min at room temperature with bromodeoxyuridine (BrdU) primary antibody (550803; BD Pharmingen) at a dilution of 1:10. After the primary antibody application, the slides were washed three times with PBS for 3 min each. Following the wash, a biotinylated secondary antibody (TP-125-BN, Lab Vision, Fremont, CA) was applied for 10 min, followed by treatment with the streptavidin-peroxidase enzyme complex (TS-125-HR, Lab Vision, Fremont, CA) for 10 min. Finally, the chromogen diaminobenzidine (DAB) (TA-125-HD, Lab Vision, Fremont, CA) was applied to visualize the immunoreaction. Mayer’s hematoxylin was used as a counterstain, and the slides stained with DAB were processed through decreasing alcohol series. After being kept in xylene for 20 min, the slides were mounted with Entellan and analyzed using the Leica DM 4000 (Germany) computer-assisted imaging system and the Leica Q LAS software [32].

### Follicle Counting and Immunopositivity Determination Method

For follicle counting and immunohistochemical assessment, ovarian tissues were sectioned serially into 4 μm thick slices. Every fifth slice was used for follicle counting, while the sixth slice was utilized for immunohistochemical evaluation. In all ovarian sections stained with Hematoxylin and Eosin (H&E), the counts of primordial, primary, and antral follicles were recorded. As part of the immunohistochemical assessment, the percentage (%) of VEGF immunostaining in each section was calculated using Image J software [33].

### Statistical Analyses

Data were entered into the SPSS 21.0 statistical software, and multiple ROC curve analyses were performed. A *p*-value of less than 0.05 was considered statistically significant.

## Results

### Experimental Animals: Rat Weight and Blood Glucose Levels

**Fig. 1:**
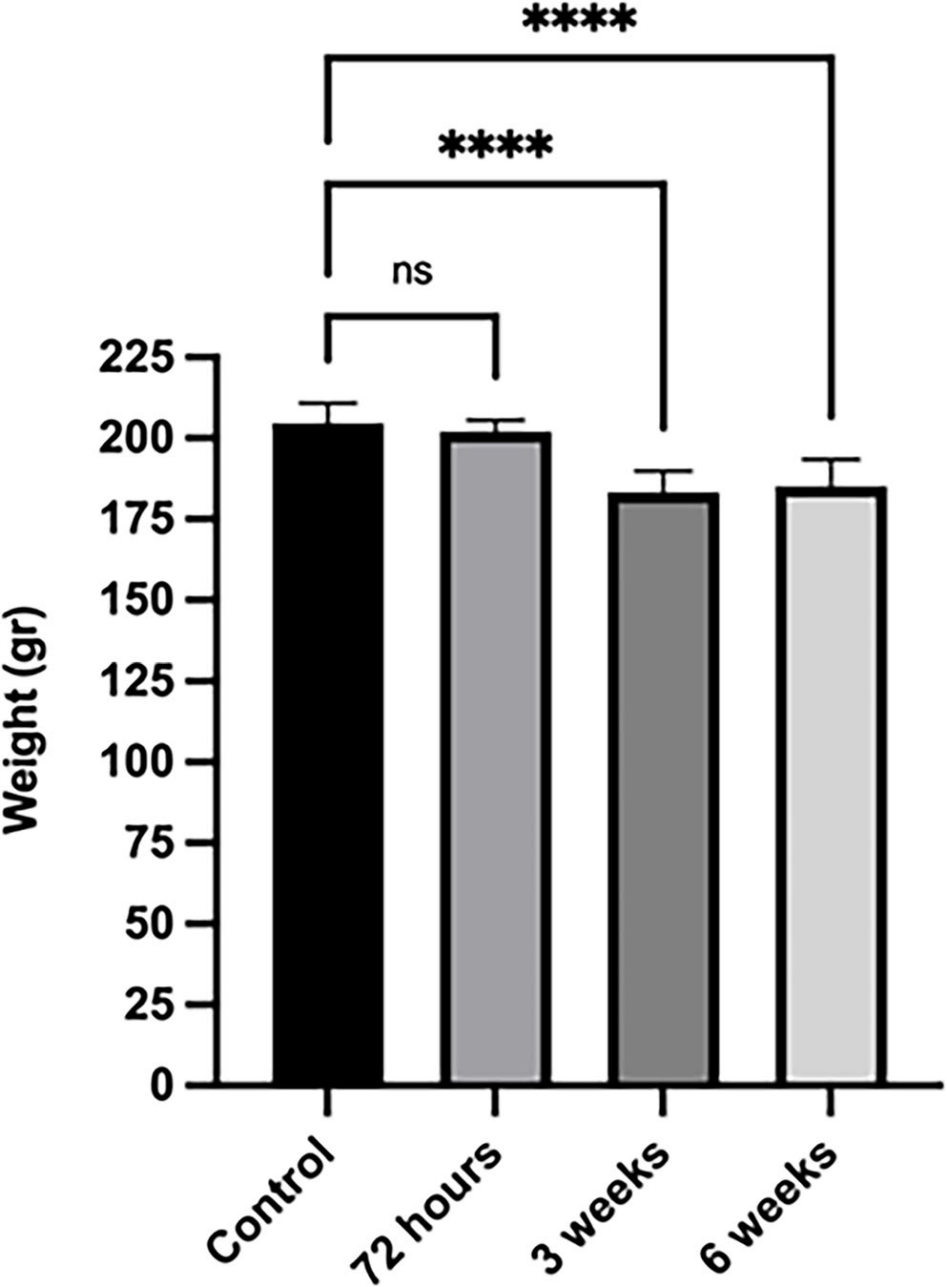
Body weight of control and streptozotocin (STZ)-treated rats at 72 h, 3 weeks, and 6 weeks. No significant change was observed at 72 h, whereas significant weight loss occurred at 3 and 6 weeks compared to the control and 72-hour groups (****p < 0.0001). Data are mean ± SEM

The initial average weight of all rats included in the study was measured at 204 g. Weight measurements taken 72 h after the STZ application did not show a significant difference compared to the baseline values. The average weight before STZ was 204 g, whereas at the 72nd hour after STZ, this value was found to be 202.5 g, with no statistically significant difference observed (*p* > 0.05). However, weight measurements taken three weeks after STZ administration showed a decrease in the average weight from the initial 204 g to 182 g. Although there was no significant difference between the groups at the end of the third week, weight loss occurred in the STZ-induced diabetic rats, as expected. At the end of the sixth week following the STZ application and after stem cell treatments, the average weight was measured at 185 g. Considering that the average weight before stem cell application was 182 g, the lack of a significant change in weight gain can be assessed as normal due to the absence of a systemic effect from the stem cell application (Fig. 1).

**Fig. 2:**
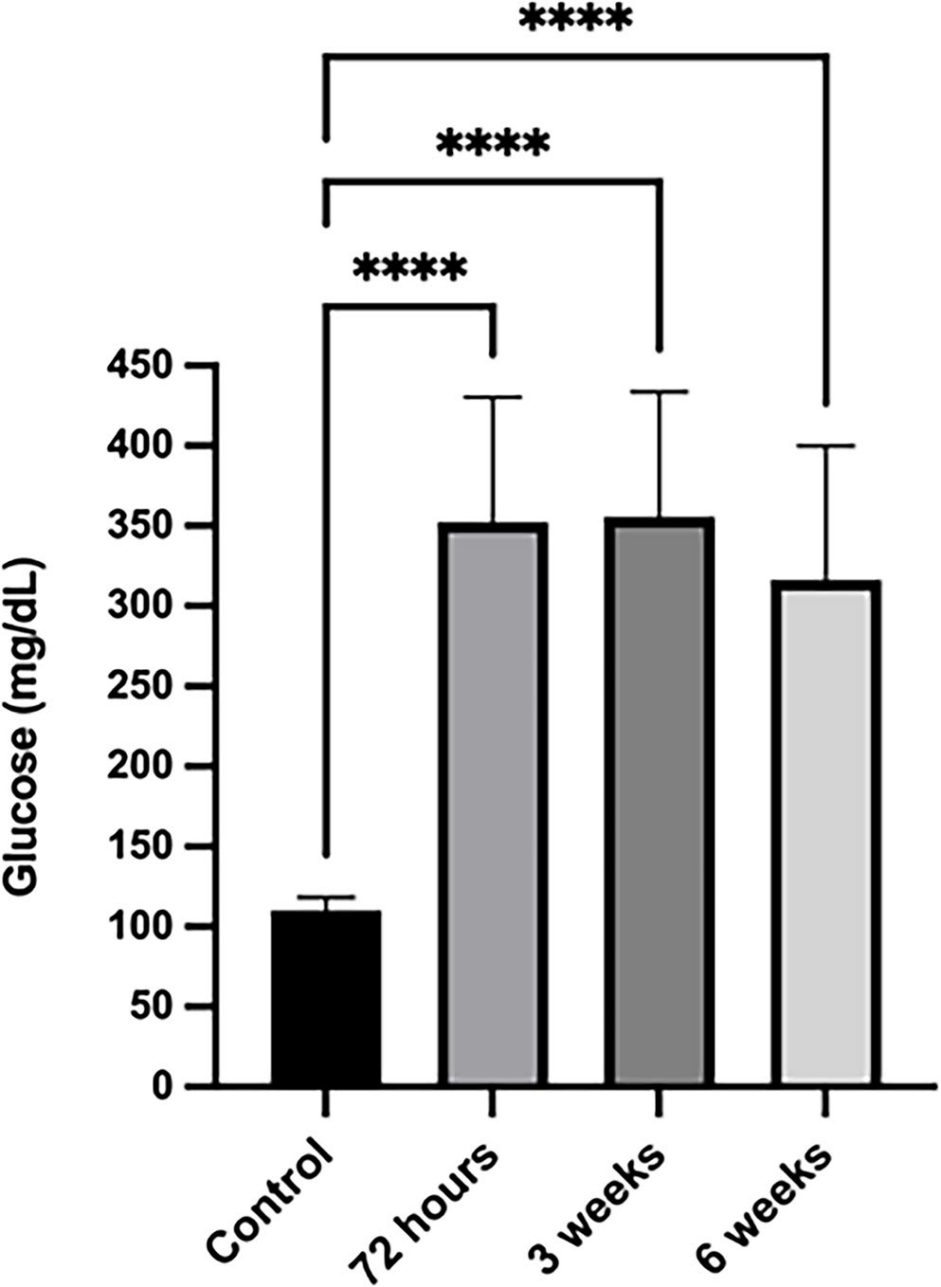
Blood glucose levels in control and streptozotocin (STZ)-treated rats at 72 h, 3 weeks, and 6 weeks. All STZ-treated groups showed significantly higher glucose levels compared to the control group (****p < 0.0001). Data are presented as mean ± SEM

The initial average blood glucose level of the rats included in the study was measured at 105 mg/dL. Blood glucose measurements taken at the 72nd hour following STZ administration showed an average value of 354 mg/dL across all groups. In measurements taken three weeks after STZ application, the average blood glucose level was 357 mg/dL, showing a considerable similarity to the values obtained at the 72nd hour. Measurements taken in the sixth week following STZ application revealed that the average blood glucose level in the diabetic groups were 359 mg/dL. (Fig. 2).

### Characterization of Mesenchymal Stem Cells

**Fig. 3:**
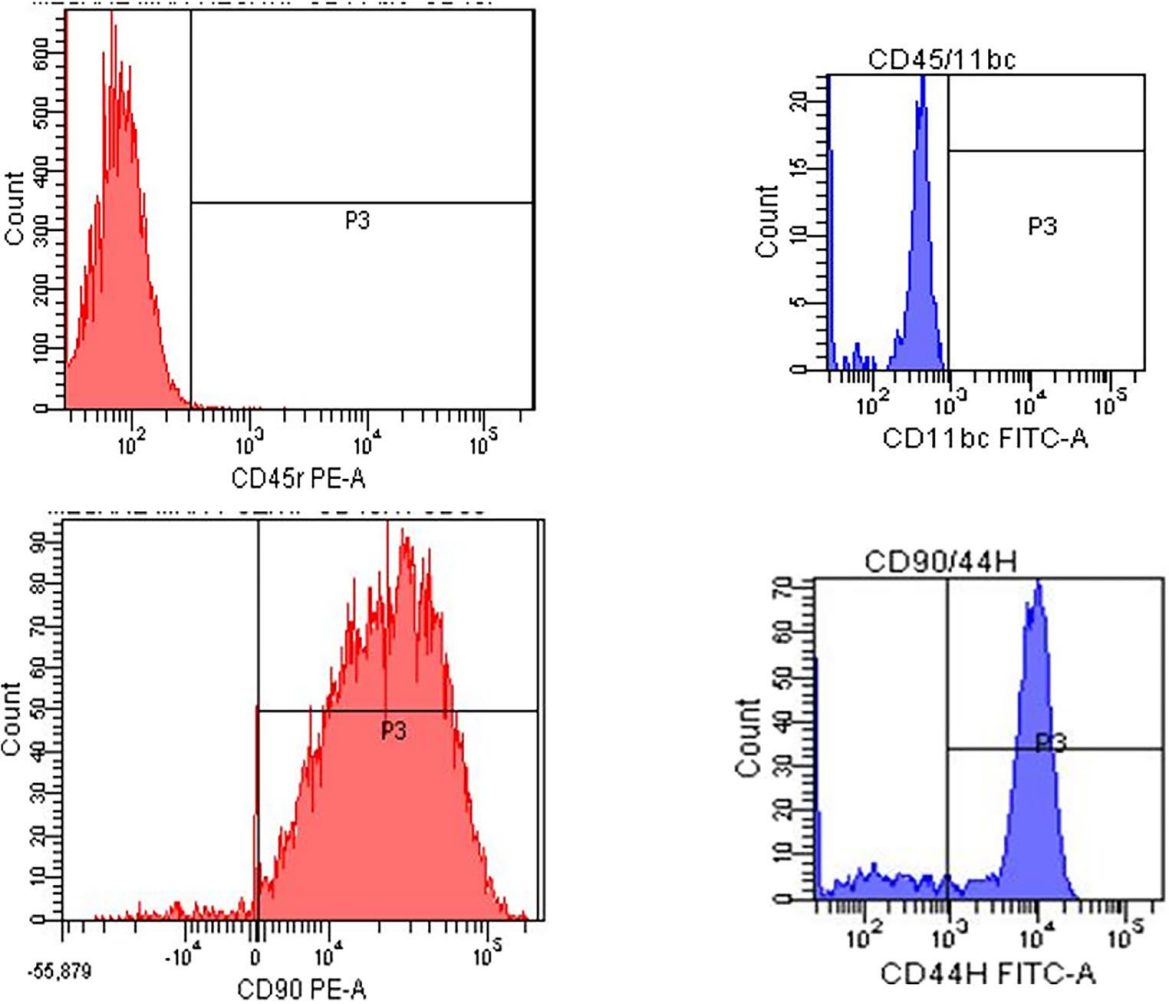
Flow cytometric characterization of mesenchymal stem cells (MSCs) isolated from rat ovary, endometrium, and bone marrow. Representative histograms show the expression of specific surface markers confirming MSC identity. Red histograms represent staining for CD45 (negative) and CD90 (positive), while blue histograms represent staining for CD11b/c (negative) and CD44 (positive). The MSCs displayed the characteristic phenotype of being negative for hematopoietic markers (CD45, CD11b/c) and positive for mesenchymal markers (CD44, CD90), in accordance with established criteria. Data are representative of three independent experiments

The establishment of mesenchymal stem cell (MSC) cultures was achieved using primary cell culture techniques, and the successful differentiation of these cells was assessed through characterization tests. These tests consisted of: (1) identification of cells based on surface markers via flow cytometry, and (2) assays demonstrating differentiation into three distinct cell types derived from the three germ layers. For this purpose, at the end of the second passage, the number and viability of the cells were determined using the Countess^®^ Automated Cell Counter (Invitrogen, USA), and then the cells were prepared in suspension for mesenchymal stem cell characterization. During immunophenotyping, CD45 (-) and CD11b/c (-) were used as negative markers, while CD44 and CD90 monoclonal antibodies were used as positive markers. The cells were transferred to various differentiation media to differentiate into adipocytes, osteocytes, and chondrocytes. The results indicated that the obtained cells possess the characteristics of mesenchymal stem cells (Fig. 3). This study facilitated the transition to differentiation studies as a second mesenchymal stem cell experiment.

**Fig. 4:**
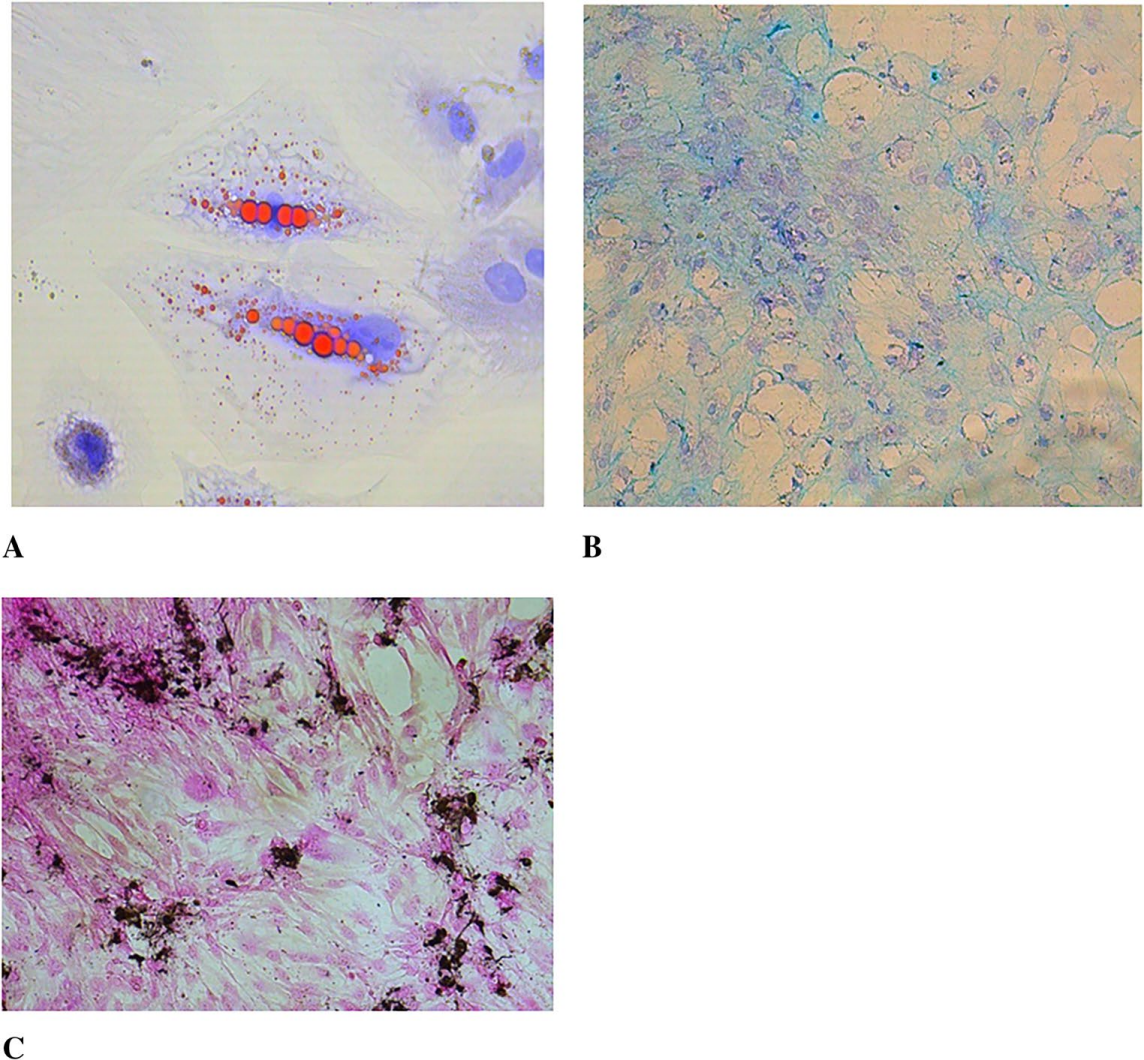
Demonstration of (**A**) Adipocyte differentiation of rat ovarian, endometrial, and bone marrow-derived mesenchymal stem cells using Oil Red staining (Leica D1000, 60x), (**B**) Chondrocyte differentiation using Alcian Blue staining (Leica D1000, 20x), (**C**) Osteocyte differentiation using Von Kossa staining (Leica D1000, 4x)

Studies were conducted using Adipocyte Differentiation Basal Medium and supplements (Gibco, USA) for the purpose of adipocyte differentiation. As a result of the experiment, adipogenic differentiation was observed through the red staining of lipid droplets, osteogenic differentiation was indicated by the black staining of calcium deposits, and chondrogenic differentiation was demonstrated by the blue staining of hyaluronic acid (Fig. 4).

### Mesenchymal Stem Cell BrdU Staining Results

**Fig. 5:**
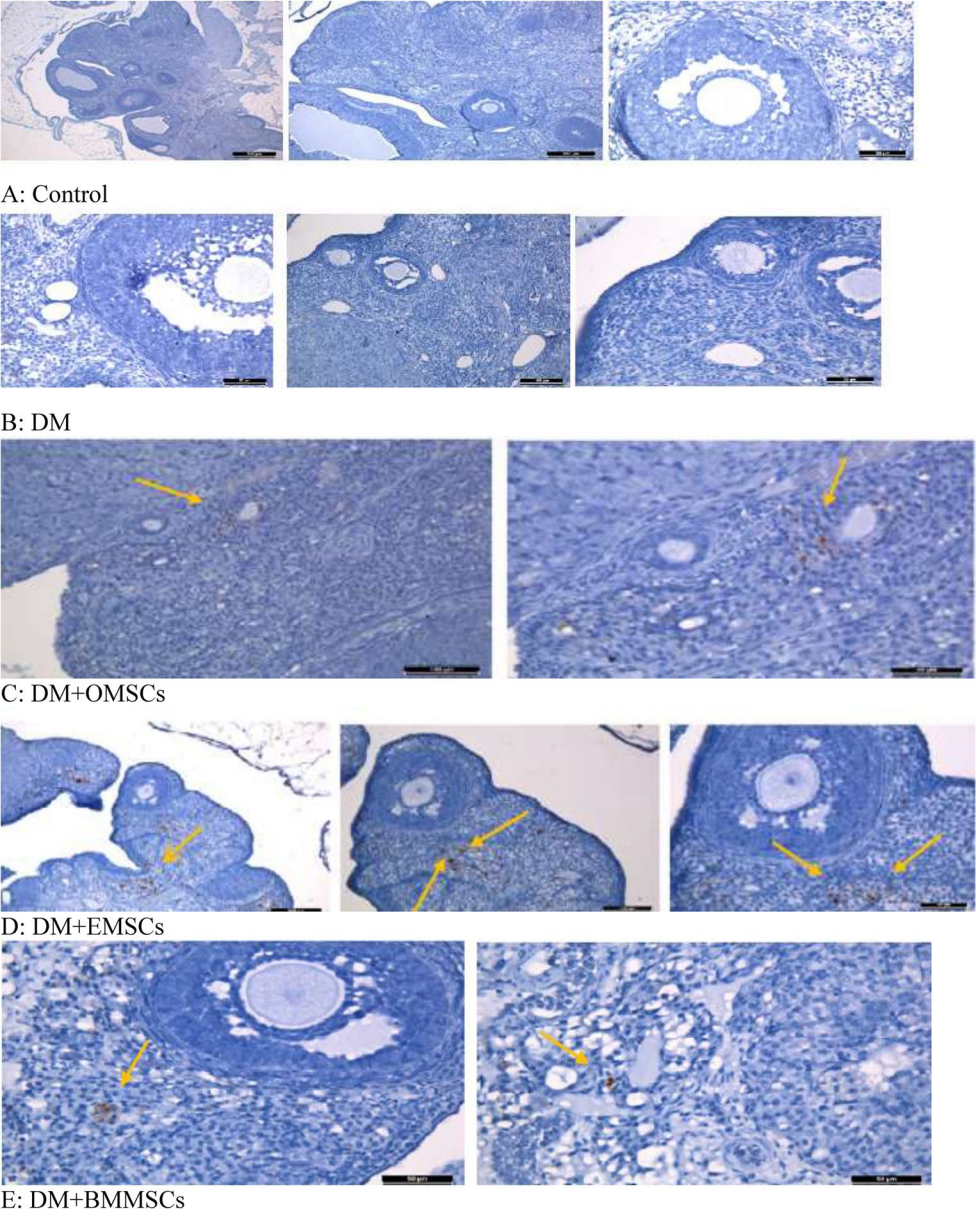
**A**: No BrdU-positive cells were detected in the control group. **B**: No BrdU-positive cells were observed in the DM group. **C**: In the DM+OMSCs group where mesenchymal stem cells were administered, BrdU immunopositivity was evaluated, revealing a widespread presence of BrdU-positive cells, particularly in the ovarian stroma and around the follicles (yellow arrows). **D**: In the DM+EMSCs group that received mesenchymal stem cells, BrdU-positive cells were also commonly found in the ovarian stroma and around the follicles (yellow arrows). **E**: In the DM+BMMSCs group where mesenchymal stem cells were administered (yellow arrows), BrdU immunopositivity was examined, showing that, similar to the other groups receiving mesenchymal stem cell treatment, BrdU positive cells were frequently located, particularly in the ovarian stroma and around the follicles

To histopathologically examine the localization of mesenchymal stem cells applied to diabetic rats at the end of the experiment and to assess their contribution to healing, these cells were stained with BrdU. No BrdU-positive cells were observed in the control and DM groups. In the DM+OMSCs, DM+EMSCs, and DM+BMMSCs groups, where mesenchymal stem cells were administered, BrdU immunopositivity was evaluated, revealing a widespread presence of BrdU-positive cells, particularly in the ovarian stroma and around the follicles in all stem cell-treated groups (Fig. 5).

### Results of Hematoxylin-eosin Staining

**Fig. 6:**
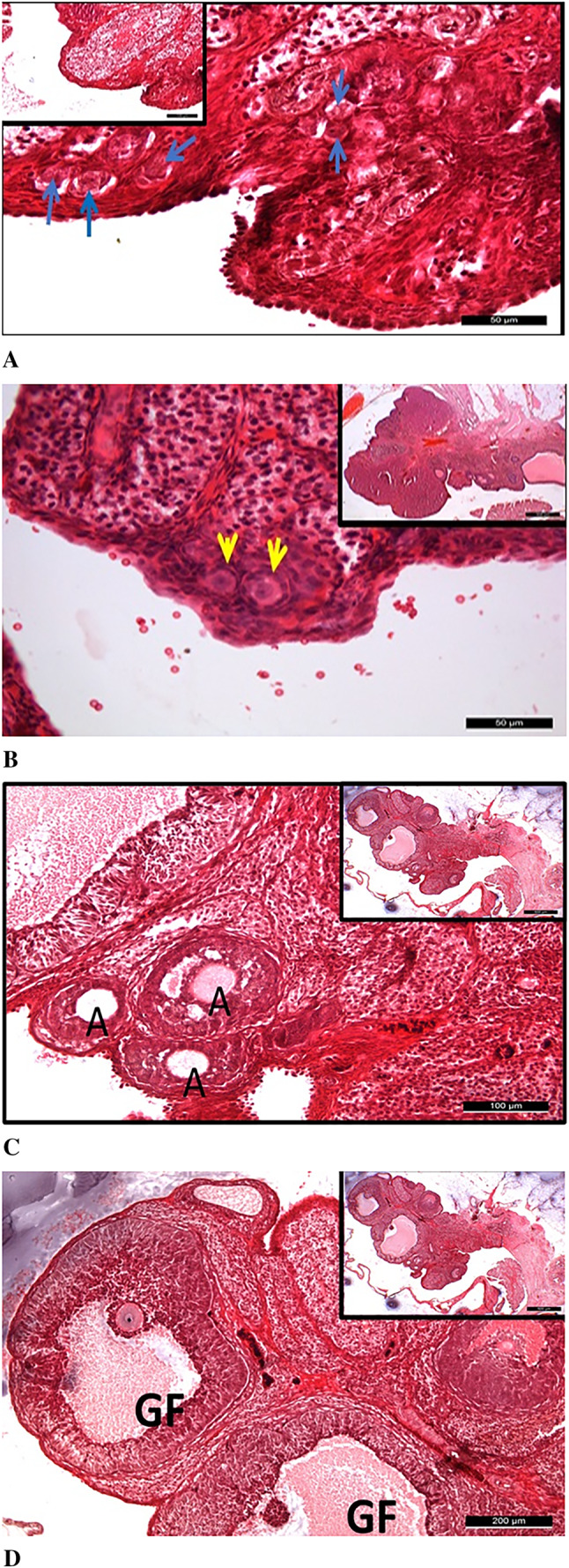
General appearance of ovarian tissue in the control group. **A**: It was observed that primordial follicles (blue arrows) in the control group maintained their normal histological structures and were numerous (main image: 50 μm, inset: 100 μm). **B**: Unilaminar primary follicles (yellow arrows) in the control group also exhibited normal histological characteristics (main image: 50 μm, inset: 500 μm). **C**: Antral follicles (AF) in the control group were found to possess a normal histological structure (main image: 100 μm, inset: 500 μm). **D**: It was determined that Graafian follicles (GF) in the control group also displayed normal histological features (main image: 200 μm, inset: 500 μm). Insets represent lower magnification views, and scale bars are indicated in each panel

As a result of the histomorphological evaluation conducted with H&E staining in this study, numerous primordial, primary, antral, and Graafian follicles, along with stromal structures, were observed in the ovarian tissue of the control group, exhibiting normal histological characteristics (Fig. 6).

**Fig. 7:**
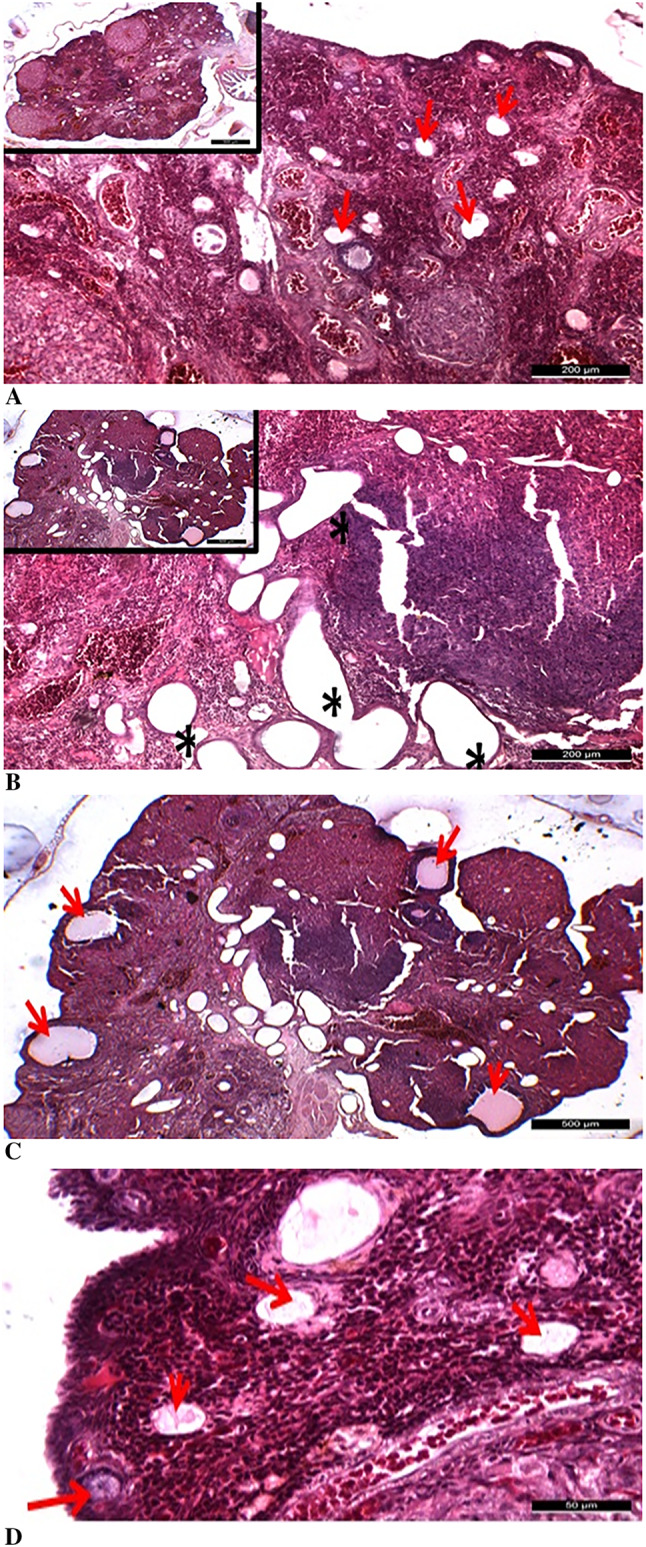
Degenerative changes observed in the diabetes group at low and high magnifications. **A**: It was determined that the follicles (red arrows) in the diabetes group underwent degeneration (200 μm). **B**: Degeneration of vascular structures was observed in the diabetes group (*) (200 μm). **C**: Irregular, degenerate Graafian follicles (red arrows) were identified in the diabetes group (500 μm). **D**: Early-stage follicles (red arrows) exhibiting degenerative changes were observed in the diabetes group (50 μm). Scale bars are indicated in each panel

In the diabetes group, numerous follicles exhibiting significant degenerative changes were observed, even at low magnifications, along with dilated vascular structures in the medullary region and occasional hematoma areas. In this group, primordial follicles were not detectable in the tissue sections, while developing follicles were noted to have thin walls due to a scarcity of follicular cell layers. These types of follicles were considered to be immature rather than regressing follicles. It was determined that the Graafian follicles present in the diabetes group also exhibited thin-walled, irregular structures and had an immature appearance (Fig. 7).

**Fig. 8:**
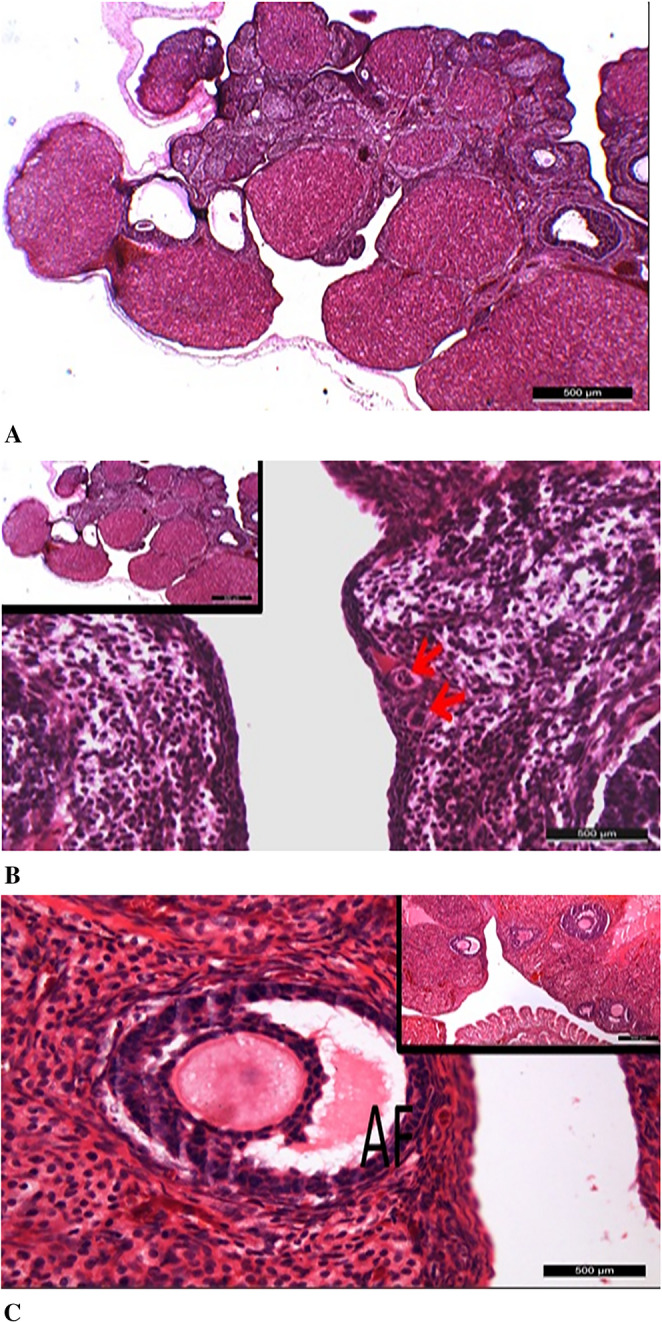
Ovarian morphology in the DM+OMSCs group. **A** – Mature and regressing Graafian follicles. **B** – Primordial follicles exhibiting normal histological structure. **C** – Antral follicles (AF) with preserved morphology. All images were captured at 500 μm, and scale bars are indicated in each panel

In the diabetic group treated with ovarian mesenchymal stem cells (DM+ OMSCs), it was observed that the number of primordial follicles decreased compared to the control group; however, they maintained their histologically normal morphology. In this group, although the number of developing follicles and antral follicles also decreased, they exhibited normal histological characteristics (Fig. 8).

**Fig. 9:**
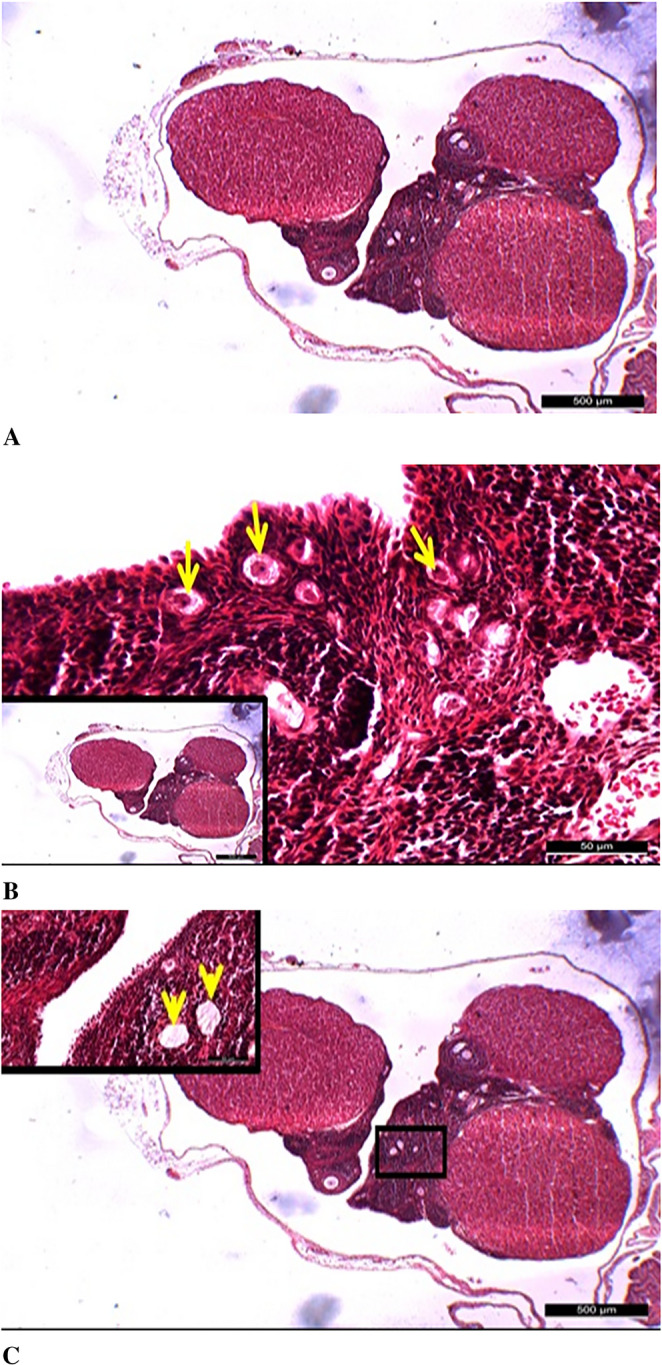
Histological features of ovarian tissue in the STZ+EMSCs group. **A** – Overall ovarian tissue with preserved histological appearance (500 μm). **B** – Numerous primordial follicles showing degeneration with prominent cytoplasmic vacuoles (50 μm). **C** – Limited degenerative dilated areas detected in the stromal tissue (500 μm). Scale bars are indicated in each panel

In the diabetic group treated with endometrial mesenchymal stem cells (DM+ EMSCs), the findings related to follicles were generally similar to those in the DM+ OMSCs stem cell group; however, widespread vacuolization in the cytoplasm of oocytes, particularly in primordial follicles, was notable. Additionally, in the endometrial stem cell group, although fewer dilated areas were observed in the stromal tissue, these findings indicated degenerative changes in the diabetic group (Fig. 9).

**Fig. 10:**
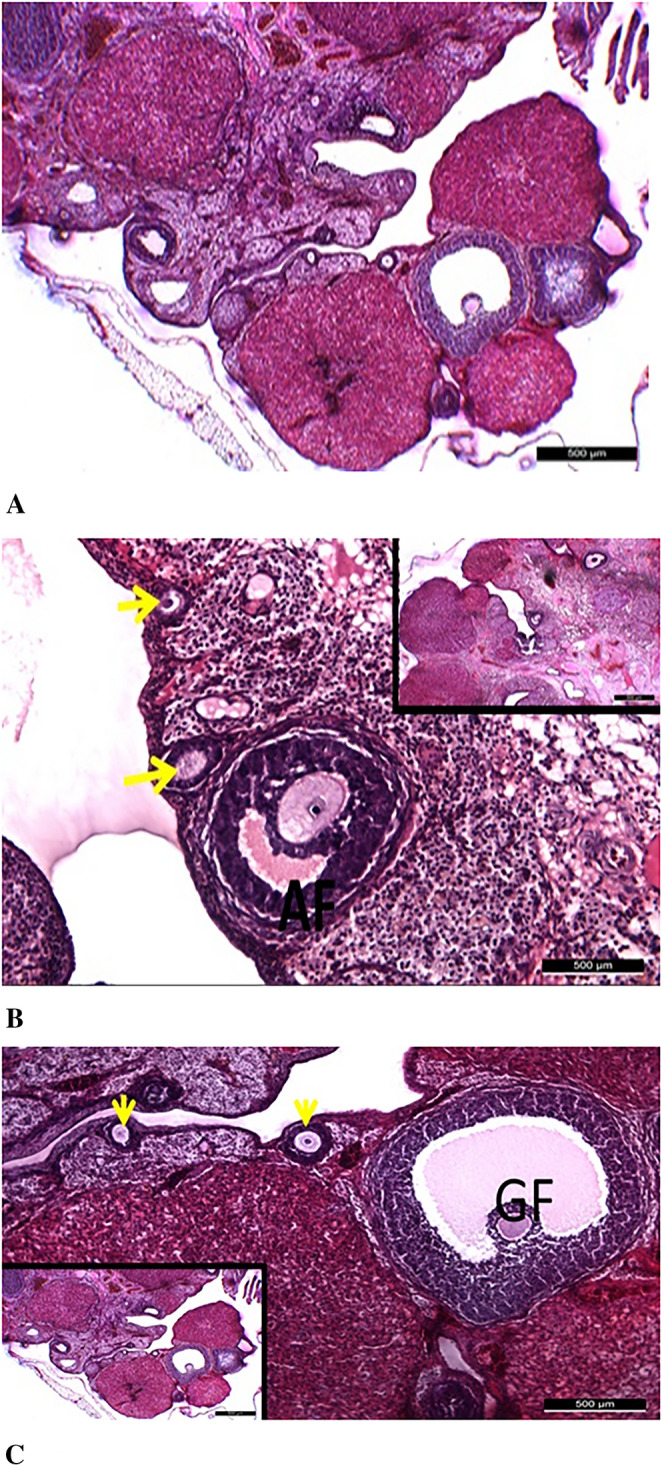
Histological examination of ovarian tissue in the DM+BMMSCs group. **A** – Ovarian tissue exhibiting a preserved histological structure. **B** – Antral and primary follicles showing normal morphology (yellow arrows). **C** – Graafian (GF) and primary follicles (yellow arrows) with typical histological features. All images were captured at 500 μm, and scale bars are indicated in each panel

In the diabetic group treated with bone marrow-derived mesenchymal stem cells (DM+ BMMSCs), findings obtained from light microscopic examinations at low and high magnifications were found to be similar to those in the DM+ OMSCs mesenchymal stem cell group. However, although the number of primordial follicles, developing follicles, and Graafian follicles decreased compared to the control group, they maintained their normal histological structure (Fig. 10).

### Immunohistochemical Staining Results

**Fig. 11:**
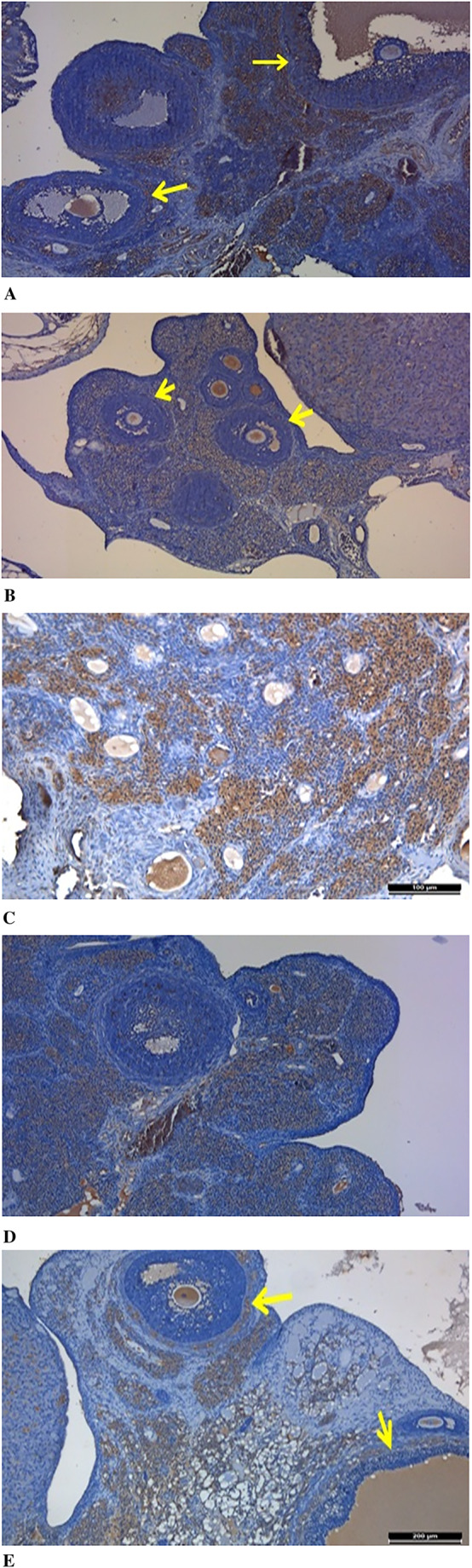
Immunohistochemical analysis of VEGF expression in ovarian tissue. **A** – Control group showing VEGF immunoreactivity (yellow arrows). **B** – DM group with VEGF immunoreactivity (yellow arrows). **C** – DM +OMSCs group (100 μm). **D** – DM +EMSCs group. **E** – DM +BMMSCs group showing VEGF immunoreactivity (yellow arrows, 200 μm). Scale bars are indicated in each panel

According to the results obtained from immunohistochemical staining, VEGF immunoreactivity was widely observed in stromal cells and in the theca layer of antral follicles in the control group. In the diabetes group, however, a weaker VEGF immunoreactivity was noted in stromal cells compared to the control group, and no immunoreactivity was detected in the theca layer surrounding the antral follicles. In the DM+OMSCs group, strong VEGF immunoreactivity was observed in stromal cells. In the DM+EMSCs group, VEGF immunoreactivity was similarly weak, observed only in stromal cells, with no immunoreactivity found in the theca layer. Findings in the DM+BMMSCs group showed similarities to the control group, although VEGF immunoreactivity in stromal cells and in the theca layer surrounding antral follicles was weaker compared to the control group. When evaluating the overall immunological results, the reactivity closest to the control group in terms of VEGF expression was found in the DM+OMSCs and DM+BMMSCs groups (Fig. 11).

### Follicle Count Results

According to the results of the multiple ROC curve analysis, the ability to discriminate between the DM, DM+OMSCs, DM+BMMSCs, and DM+EMSCs groups based on primordial, primary, antral, and VEGF (vascular endothelial growth factor) values was found to be 0.872 (AUC, area under the curve; *p* = 0.002), 0.818 (AUC; *p* = 0.008), 0.727 (AUC; *p* = 0.058), and 0.984 (AUC; *p* < 0.001), respectively (Table 1).

**Table 1:** ROC curve analysis results for follicle counts and VEGF values

Variable	AUC	Standard Error (AUC)	*p*-value	95% Confidence Interval for AUC	
				Lower Limit	Upper Limit
Primordial	0,872	0,069	0,002	0,738	1
Primary	0,818	0,080	0,008	0,66	0,975
Antral	0,727	0,114	0,058	0,503	0,95
VEGF	0,984	0,018	< 0,001	0,949	1

*AUC*, area under the curve; *VEGF*, vascular endothelial growth factor; *DM*, diabetes mellitus; *OMSCs*, ovarian mesenchymal stem cells; *BMMSCs*, bone marrow–derived mesenchymal stem cells; *EMSCs*, endometrial mesenchymal stem cells

When the number of primordial follicles was statistically evaluated across all groups, a significant reduction was observed in all groups compared to the control group. Upon analysis of primordial follicle counts in the diabetes group (DM group), it was determined that the treatment groups exhibited an increase in the number of primordial follicles compared to the DM group. This increase was statistically significant in all treatment groups except the DM+EMSCs group. The differences between the DM+OMSCs and DM+EMSCs groups, as well as between the DM+EMSCs and DM+BMMSCs groups, were statistically significant, while no significant difference was identified between the DM+OMSCs and DM+BMMSCs groups. Among the treatment groups, the DM+BMMSCs group demonstrated the highest number of primordial follicles.

When the number of primary follicles was statistically evaluated across all groups, a decrease was observed in the treatment groups compared to the control group. This reduction was statistically significant in all groups except for the DM+OMSCs group when compared with the control group. The analysis of primary follicle counts in the diabetes group (DM) revealed an increase in antral follicle numbers in the treatment groups compared to the diabetes group. This increase was statistically significant in all treatment groups except for the DM+EMSCs group. The differences between the DM+OMSCs and DM+EMSCs groups, as well as between the DM+EMSCs and DM+BMMSCs groups, were statistically significant, while no significant difference was observed between the DM+OMSCs and DM+BMMSCs groups. Among the treatment groups, the DM+OMSCs group was identified as having the highest number of primary follicles.

**Fig. 12:**
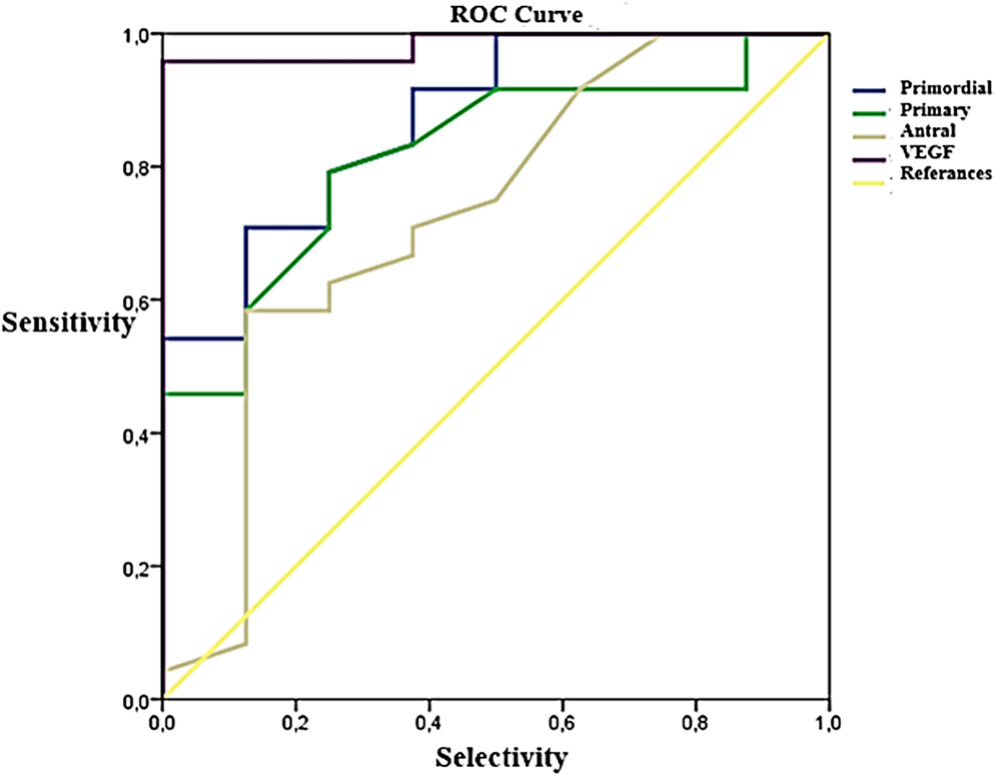
Receiver operating characteristic (ROC) curve analysis of follicle counts and VEGF values across experimental groups. The diagnostic performance of different parameters was evaluated for discrimination between the DM, DM+OMSCs, DM+BMMSCs, and DM+EMSCs groups. The ROC curves represent primordial follicles (dark blue), primary follicles (green), antral follicles (brown), and VEGF (purple), while the yellow line indicates the reference diagonal (no-discrimination line). The area under the curve (AUC) values were calculated as 0.872 for primordial (*p* = 0.002), 0.818 for primary (*p* = 0.008), 0.727 for antral (*p* = 0.058), and 0.984 for VEGF (*p* < 0.001), indicating that VEGF and primordial follicle counts provided the strongest discriminatory power among the groups

When the number of antral follicles was statistically evaluated across all groups, a reduction in antral follicle numbers was observed in all treatment groups compared to the control group. This reduction was found to be statistically significant in all groups except for the DM+BMMSCs group when compared with the control group. Upon examining the number of antral follicles in the diabetes group (DM), an increase in antral follicle numbers was observed in all treatment groups compared to the DM group. However, when comparing the diabetes group with the treatment groups, this increase was not statistically significant. No statistically significant differences were found between the DM+OMSCs and DM+EMSCs groups, the DM+OMSCs and DM+BMMSCs groups, or the DM+EMSCs and DM+BMMSCs groups. Among the treatment groups, the DM+BMMSCs group had the highest number of antral follicles (Fig. 12).

## Discussion

In this study, the therapeutic effects of stem cells derived from different sources on the damage caused by diabetes in the ovaries were investigated in rats with experimentally induced diabetes via STZ. The results of weight measurements, blood glucose levels, and immunohistopathological evaluations in diabetic rats were compared with findings in the existing literature.

In this study, 45 mg/kg of STZ was administered intraperitoneally to adult female rats in the experimental groups, compared to the control group (normal group with no intervention). Seventy-two hours after the administration, rats with blood glucose levels of 200–250 mg/dL or higher, as measured from tail vein blood samples, were considered diabetic. In the third week of the experiment, stem cells obtained from different sources were administered to the experimental animals, and at the end of the sixth week, the rats were sacrificed, and ovarian tissues were extracted.

Histopathological examinations revealed that the diabetic group without stem cell therapy showed significant follicular degeneration, stromal degeneration, stromal fibrosis, and increased inflammation in the ovarian tissue compared to the control group. Moreover, morphological disruptions in the ovarian structure were observed in the diabetic group. In contrast, examination of the ovarian tissue in the control group showed that numerous primordial, primary, antral, and Graafian follicles, as well as stromal structures, preserved their normal histological features. These findings were consistent with the study conducted by Pala et al. in 2013 [11].

In the diabetic group, numerous degenerative changes were observed in the follicles, along with prominently dilated vascular structures and occasional hematoma areas in the medullary region. In this group, no primordial follicles were detected in the sections, and the developing follicles were identified as having thin walls. This condition was associated with a reduced number of follicular cell layers, and these follicles were thought to be immature rather than regressing follicles. Additionally, the examinations revealed that the Graafian follicles appeared as thin-walled, irregular, and immature structures. Experimental studies have shown that mesenchymal stem cells labeled by different methods can reach the theca layers of the ovarian follicles [34, 35]. In our study, BrdU-labeled stem cells were also found to localize within the ovarian tissue and around the follicles in all three cell groups.

In the groups treated with stem cells, although the number of developing and antral follicles was lower compared to the control group, they exhibited a normal histological structure. The primordial follicle values were determined as follows: 79.38% in the control group, 20.88% in the diabetes group, 47.13% in the DM+OMSCs group, 24.13% in the DM+EMSCs group, and 53.38% in the DM+BMMSCs group. In the DM+OMSCs group, although the number of primordial follicles was lower compared to the control group, the ovarian tissue exhibited a normal histological structure. These values were recorded as 79.38% in the control group and 47.13% in the DM+OMSCs group, respectively. In this group, although the number of developing and antral follicles was lower, they exhibited a normal histological structure. These values were determined as 24.50% in the control group and 14.75% in the DM+OMSCs group.

The findings in the Dm+EMSCs group were generally similar to those in the DM+ OMSCs group; however, widespread vacuolizations in the oocyte cytoplasm of primordial follicles were particularly notable. The number of primordial follicles in this group was determined to be 24.13%, and dilated areas and signs of degeneration in the stroma, similar to the diabetic group without stem cell therapy, were observed. In this group, the developing and antral follicles were also fewer in number compared to the control group; these values were recorded as 24.50% in the control group and 12.00% in the DM+OMSCs group, respectively.

In the DM+BMMSCs mesenchymal stem cell group, light microscopic examinations revealed findings similar to those in the DM+OMSCs group. Despite the fact that the number of primordial, developing, and Graafian follicles was lower compared to the control group, they displayed features similar to normal histological structures. These values were recorded as 79.38% in the control group and 53.38% in the DM+BMMSCs group. Additionally, the number of developing and antral follicles in this group was also lower compared to the control group, with values recorded as 24.50% in the control group and 17.50% in the DM+OMSCs group.

In the experimental studies conducted by Beşikçioğlu et al. in 2019 on rats, cyclophosphamide was administered on the first day, followed by stem cell application. The researchers terminated the experiment on the 10th day following the initial damage and examined the tissues. Histopathological examinations revealed that ovarian stromal-derived mesenchymal stem cells had a greater protective effect on ovarian tissue compared to bone marrow-derived mesenchymal stem cells [20]. In our study, mesenchymal stem cells from three different sources were used to repair diabetes-induced damage in ovarian tissue. It was found that ovarian-derived mesenchymal stem cells and bone marrow-derived mesenchymal stem cells provided more effective damage repair compared to endometrium-derived mesenchymal stem cells.

In the mouse studies conducted by Sun et al. in 2013 using cyclophosphamide, adipose-derived mesenchymal stem cells were administered both to the ovarian tissue and intravenously 15 days after cyclophosphamide administration. Following the application, half of the mice were sacrificed after 1 week, and the other half after 1 month, with their ovarian tissues collected for analysis [36]. In our study, unlike the study by Sun and colleagues, mesenchymal stem cells from three different sources were used. The effectiveness of bone marrow-derived and ovarian tissue-derived mesenchymal stem cells in repairing ovarian damage resulting from chronic diabetes was demonstrated.

In the study conducted by Terraciano et al. in 2014, ovarian damage was induced in mice using cisplatin. One week after the induced damage, adipose-derived stem cells, female germline-derived stem cells, and cells obtained from ovarian suspension were administered in a 1 × 10^4^ / 5 µL PBS solution. Following the cell administration, half of the mice were euthanized on the 7th day, while the other half were euthanized on the 14th day for examination of ovarian tissues. The results indicated that adipose-derived stem cells and female germline-derived stem cells eliminated the ovarian damage caused by cisplatin; however, the application of cells obtained enzymatically from ovarian tissue was found to be ineffective [37].

In our study, unlike the work of Terraciano et al., it was observed that bone marrow-derived and ovarian-derived mesenchymal stem cells obtained via the explant method were effective in repairing ovarian damage caused by diabetes, a chronic systemic disease. Wang et al. treated cisplatin-induced ovarian damage in mice in 2013 using stem cells derived from umbilical cord blood. These stem cells were injected into the ovaries in a solution of 10^7^ cells/mL/100 µL PBS. Forty-eight hours after the stem cell application, unlike our experimental model, an intraperitoneal injection of 5 µL hCG was administered. One week after the cell application, ovarian tissues were excised for histopathological examination. The results demonstrated that umbilical cord-derived stem cells improved cisplatin-induced ovarian damage [38]. Furthermore, in some clinically reported cases, it has been noted that premature ovarian insufficiency caused by cisplatin or similar chemotherapeutic agents improved following bone marrow-derived hematopoietic stem cell transplants performed for various reasons, leading to spontaneous pregnancies [39–40].

## Conclusion

In reproductive medicine, stem cells are utilized for the preservation of fertility and the production of functional gamete cells from stem cells. In this study, we investigated the therapeutic effects of stem cells. Stem cells support tissue repair through endocrine and paracrine effects by creating a microenvironment through the cytokines they secrete. Additionally, due to their ability to differentiate into various cell types, they contribute to tissue regeneration.

As a result, in this study, we developed an approach to repair ovarian damage caused by diabetes, a chronic disease prevalent in society, using mesenchymal stem cells derived from bone marrow, ovarian tissue, and endometrium in a rat model. When evaluating the results of the study and discussing them in conjunction with the relevant literature, it was observed that various stem cell sources could be effective in repairing ovarian damage, particularly that the bone marrow and ovarian-derived mesenchymal stem cells included in our study may serve as cellular therapeutic products. These findings should be supported by studies involving larger sample sizes and various gene expression and pathway analyses to better understand the potential mechanisms of tissue restoration and regeneration.
